# Inflammation and ER Stress Downregulate BDH2 Expression and Dysregulate Intracellular Iron in Macrophages

**DOI:** 10.1155/2014/140728

**Published:** 2014-12-01

**Authors:** Susu M. Zughaier, Brandon B. Stauffer, Nael A. McCarty

**Affiliations:** ^1^Department of Microbiology and Immunology, Veterans Affairs Medical Center (VAMC (151-I)), Emory University School of Medicine and Children's Healthcare of Atlanta, 1670 Clairmont Road, Atlanta, GA 30033, USA; ^2^Emory-Children's Center for Cystic Fibrosis and Airways Disease Research, Division of Pulmonology, Allergy/Immunology, Cystic Fibrosis and Sleep, Department of Pediatrics, Emory University School of Medicine and Children's Healthcare of Atlanta, Atlanta, GA 30322, USA

## Abstract

Macrophages play a very important role in host defense and in iron homeostasis by engulfing senescent red blood cells and recycling iron. Hepcidin is the master iron regulating hormone that limits dietary iron absorption from the gut and limits iron egress from macrophages. Upon infection macrophages retain iron to limit its bioavailability which limits bacterial growth. Recently, a short chain butyrate dehydrogenase type 2 (BDH2) protein was reported to contain an iron responsive element and to mediate cellular iron trafficking by catalyzing the synthesis of the mammalian siderophore that binds labile iron; therefore, BDH2 plays a crucial role in intracellular iron homeostasis. However, BDH2 expression and regulation in macrophages have not yet been described. Here we show that LPS-induced inflammation combined with ER stress led to massive BDH2 downregulation, increased the expression of ER stress markers, upregulated hepcidin expression, downregulated ferroportin expression, caused iron retention in macrophages, and dysregulated cytokine release from macrophages. We also show that ER stress combined with inflammation synergistically upregulated the expression of the iron carrier protein NGAL and the stress-inducible heme degrading enzyme heme oxygenase-1 (HO-1) leading to iron liberation. This is the first report to show that inflammation and ER stress downregulate the expression of BDH2 in human THP-1 macrophages.

## 1. Introduction

Iron is an essential nutrient required for numerous cell functions and in order to avoid metal toxicity iron metabolism is tightly regulated [1]. Altered iron homeostasis and dysregulated cellular iron metabolism are associated with many chronic diseases including diabetes mellitus, cardiovascular diseases, arthritis, and neurodegenerative diseases. Altered iron homeostasis in chronic inflammation is characterized by misdistribution of iron that is manifested in hypoferremia, commonly called anemia of chronic inflammation, and iron retention in macrophages and the reticuloendothelial system [2, 3].

Macrophages play a central role in iron homeostasis by uptake of damaged and senescent red blood cells and recycle large amounts of iron daily [4, 5]. Macrophages also play a very important role in host defense by detecting invading pathogens and responding in order to clear infection. Since iron is essential for bacterial growth, macrophages retain iron to limit its extracellular bioavailability which thereby limits bacterial growth. This response to infection is called the iron-limiting innate immune defense mechanism. Hepcidin is the master iron regulating hormone that limits iron egress from macrophages [6]. Hepcidin binds to ferroportin, the only known iron exporter, leading to its internalization and degradation and consequent iron retention [7]. Therefore, cellular iron homeostasis and iron egress via the hepcidin-ferroportin axis are very tightly regulated during infection and inflammation [8–11].

Iron is a highly reactive metal; thus, to reduce metal toxicity, iron is maintained bound to hemoproteins and in iron-sulfur clusters or safely stored in ferritin cages [1]. Free intracellular iron or poorly liganded iron is toxic since it catalyzes the production of reactive oxygen species (ROS). Therefore, macrophages detoxify labile iron by multiple mechanisms. Recently, the presence of a mammalian siderophore, an iron-chelating small molecule, was reported and the iron-binding moiety of this molecule was identified as 2,5-dihydroxybenzoic acid (2,5DHBA) [12]. This mammalian siderophore captures free cytosolic iron and thus protects the cells from oxidant stress [12]. The enzyme that catalyzes the synthesis of 2,5DHBA is a member of the short-chain dehydrogenase/reductase family (DHRS6) [13] called 3-hydroxybutyrate dehydrogenase type 2 (BDH2) [12]. Inhibition of BDH2 expression in murine pro-B lymphocytic cells led to the depletion of the mammalian siderophore and the accumulation of labile iron. The physiological role of BDH2 in sensing cellular iron was further confirmed by the discovery that the BDH2 enzyme contains an iron responsive element that interacts with iron regulatory proteins that control posttranscriptional gene expression [14]. Recently, Zughaier et al. reported that BDH2 expression in macrophages is suppressed upon bacterial infection suggesting an important role for BDH2 in iron-limiting innate immunity [15]. More recently, the Devireddy group further confirmed the role of BDH2 in cellular iron homeostasis and showed that depletion of the endogenous siderophore 2,5-DHBA in a mouse model resulted in microcytic anemia and iron overload in the spleen [16, 17]. They also reported that BDH2 and ferritin-H synergistically regulate intracellular iron levels in an* in vitro* experimental cell culture model [18]. Therefore, BDH2 is required for intracellular iron homeostasis [12]. However, the role of BDH2 in iron-limiting innate immune defenses in macrophages is not known.

Macrophages engulf senescent RBCs and recycle hemoglobin; thus, they express heme oxygenase-1 (HO-1), the catalytic enzyme that degrades heme, consequently liberating iron and producing carbon monoxide [19]. HO-1 is highly induced in response to inflammation, oxidative stress, and hypoxia [19, 20] due to its cytoprotective effects and anti-inflammatory activity [21, 22]. Therefore, HO-1, as a stress-inducible protein, is an important player in intracellular iron homeostasis. Further, neutrophil gelatinase-associated lipocalin (NGAL) [23] functions as an iron carrier protein that shuttles bound iron across tissues by internalization with a membrane-localized receptor [24, 25] and also plays a critical physiological role in intracellular iron homeostasis by trafficking the iron-bound siderophore 2,5DHBA [12] and iron-catecholamine complexes [26]. NGAL plays a crucial role in iron-limiting innate immune defenses since it limits iron availability by also scavenging bacterial siderophores and thereby exerts an antibacterial function [27–29].

Intracellular iron homeostasis is disrupted by endoplasmic reticulum (ER) stress [30]. ER stress induced upon cellular perturbations is regulated by activation of the unfolded protein response to restore homeostatic cellular functions [30]. Control of ER stress is required for cell survival and uncontrolled ER stress leads to cell death [31, 32]. ER stress alters cellular iron homeostasis by inducing hepcidin expression [33, 34]. Altered iron homeostasis and ER stress are associated with chronic neurodegenerative diseases such as Huntington's disease and Alzheimer's disease [35, 36]. It is not known whether ER stress also affects BDH2 expression. The goal of this study was to investigate whether ER stress and inflammation modulate BDH2 expression in macrophages.

Here, we show that LPS-induced inflammation combined with chemically induced ER stress led to BDH2 downregulation, increased the expression of ER stress markers including CHOP, upregulated hepcidin expression, downregulated ferroportin expression, caused iron retention in macrophages, and dysregulated cytokine release from macrophages. Further, we show that ER stress combined with inflammation dramatically upregulated NGAL expression as well as the inducible heme oxygenase-1 enzyme in macrophages. We also show that vitamin D treatment prior to LPS restored BDH2 expression. This is the first report to show that ER stress and inflammation downregulate the expression of BDH2 in human THP-1 macrophages. The results suggest that BDH2 plays an important role in modulating iron-limiting innate immune defenses.

## 2. Materials and Methods

### 2.1. Reagents

RPMI 1640 medium, Dulbecco's modified Eagle medium (D-MEM), HBSS, fetal bovine serum (FBS), penicillin/streptomycin, sodium pyruvate, and nonessential amino acids were obtained from Cellgro Mediatech (Herdon, VA). Magnetic beads for a panel of human cytokines and chemokines were from Invitrogen (Carlsbad, CA). Tunicamycin, calcein-AM, and thiazolyl blue tetrazolium bromide were purchased from Sigma Aldrich (St. Louis, MO). Lipopolysaccharide [37] from* Neisseria meningitidis* serogroup B was purified and quantified as previously described [38].

### 2.2. Cell Culture and Macrophage Stimulation

THP-1 macrophage-like cells were grown in RPMI 1640 medium supplemented with 10% FBS, 50 *μ*g/mL penicillin, and 50 IU/mL of streptomycin. HEK293 cells were grown in D-MEM supplemented with 10% FBS, 50 *μ*g/mL penicillin, and 50 IU/mL of streptomycin. Freshly grown cells were harvested and adjusted to 1 million cells/mL and then transferred into 12-well tissue culture plates at 2 mL/well. To induce ER stress cells were treated with tunicamycin (10 *μ*g/mL in DMSO) and incubated overnight at 37°C. Control cells were treated with DMSO only and incubated similarly. Following overnight exposure to tunicamycin, cells were exposed to LPS (40 ng/mL) to induce inflammation and incubated for 6 hr at 37°C. Cells were centrifuged and supernatants were saved at −20°C for cytokine measurements. Harvested cells were washed with PBS and then placed in RLT buffer (Qiagen; Hilden, Germany) containing 1% *β*-mercaptoethanol, passed over QiaShredder columns, and the resulting lysates were saved at −80°C for mRNA extraction.

### 2.3. RNA Isolation, Quantitative Real-Time PCR, and Gene Expression Analysis

RNA was isolated using RNeasy Mini kits (Qiagen) following the manufacturer's instructions as previously described [39]. Briefly, cell lysates saved in RLT buffer were mixed in 70% ethanol and then passed over RNeasy columns. Columns were washed and treated with 10 *μ*L of RNase-free DNase (Qiagen) for 15 min at room temperature prior to RNA extraction, followed by additional washing and centrifugation. RNA was eluted in 35 *μ*L of RNase-free water and then was reverse transcribed to cDNA using QuantiTect Reverse Transcription kit (Qiagen) following the manufacturer's instructions. Relative gene expression was determined by quantitative RT-PCR performed on resulting cDNA using SYBR Green (Promega; Madison, WI) following the manufacturer's instructions. The mRNA level was calculated in reference to *β*-actin and fold change gene expression was calculated in reference to controls using the ΔΔCT method. Results were normalized to unstimulated DMSO-treated cells which were used as controls for basal gene expression level. The following primers were used for qRT-PCR reactions: human hepcidin 5′-GACCAGTGGCTCTGTTTTCC-3′ and 5′-CACATCCCACACTTTGATCG-3′; human BDH2 5′-CAGCGTCAAAGGAGTTGTGA-3′ and 5′-TGGCGTATCAACTGTTCCTG-3′; human NGAL 5′-ATGACATGAACCTGCTCGATA-3′ and 5′-TCATAGTCGTTCATTATCTTC-3′; human *β*-actin 5′-TCTTCCAGCCTTCCTTCCT-3′ and 5′-AGCACTGTGTTGGCGTACAG-3′. Primers for ER stress markers used in this study are as follows: human hsXBP-1 5′-TTCCGGAGCTGGGTATCTCA-3′ and 5′-GAACCCCCGTATCCACAGTC-3′; human GRP78 5′-ACGGCAGCTGCTATTGCTTA-3′ and 5′-TCCTGACATCTTTGCCCGTC-3′; human CHOP 5′-GACCTGCAAGAGGTCCTGTC-3′ and 5′-GCAGGGTCAAGAGTGGTGAA-3′. Ferroportin (Hs_SLC40A1_1_SG), transferrin receptor (Hs_TFRC_1_SG), and heme oxygenase (Hs_HMOX1_1_SG) QuantiTect primers were purchased from Qiagen.

### 2.4. Western Blot Analysis

Freshly grown THP-1 cells were harvested and adjusted to 1 million cells/mL and 8 mL of cells was transferred into 6-well tissue culture plates. To induce ER stress, cells were treated with tunicamycin (10 *μ*g/mL) and incubated overnight at 37°C. Control cells were treated with DMSO only and incubated similarly. Following overnight incubation, some cells were exposed to LPS (40 ng/mL) to induce inflammation and incubated for 6 hr at 37°C. Harvested THP-1 cells were lysed in RIPA buffer (Boston BioProducts; Boston, MA) and centrifuged to remove nuclei and debris, and the protein concentration of the supernatant was measured using a BCA protein assay (Thermo; Waltham, MA). Equal amounts of protein were removed from each macrophage treatment group, mixed with 6X Laemlli buffer, and boiled for 15 minutes. For untransfected HEK lysate and the BDH2 transfected HEK positive control groups (Origene Technologies; Rockville, MD), 50 ng of protein was loaded to adjust for overexpression. Samples were loaded onto a 12% MiniProtean TGX gel (BioRad; Hercules, CA), electrophoresed, and transferred onto nitrocellulose membranes (BioRad; Hercules, CA). To detect BDH2, membranes were incubated with anti-BDH2 antibody (TrueMAB antibody clone 2G1 from Origene). To detect actin, membranes were incubated with a mouse anti-*β* actin antibody (Sigma). In both cases, membranes were incubated with an HRP-conjugated anti-mouse secondary antibody (Cell Signaling; Danvers, MA), incubated in Amersham western blot detection reagent (GE Healthcare; Cleveland, OH), and visualized with HyBlot CL autoradiography film (Denville Scientific; Metuchen, NJ).

### 2.5. Cytokine and Chemokine Measurements

Cytokines and chemokines released from THP-1 macrophages treated with tunicamycin alone, LPS alone, or both tunicamycin and LPS as described above were measured using Luminex magnetic beads (Invitrogen) following the manufacturer's instructions.

### 2.6. Measurement of Iron Retention in Macrophages

Intracellular accumulation of labile iron in macrophages was measured using the well-established calcein-AM method [40]. Freshly grown THP-1 cells were treated with tunicamycin (10 *μ*g/mL) and incubated overnight prior to stimulation with LPS (40 ng/mL) for 6 hr. Control cells were treated with DMSO only. Harvested cells were resuspended in RPMI 1640 medium supplemented with 10% FBS, and 0.5 *μ*M calcein-AM was added and incubated for 15 min at 37°C. Calcein-loaded cells were washed twice with PBS to remove extracellular calcein-AM and resuspended in HBSS. 100 *μ*L aliquots, containing 0.25 million cells, were transferred into quadruplicate wells in black 96-well plates. After a 20 min incubation at 37°C, fluorescence was determined at 485 nm (excitation) and 535 nm (emission) wavelengths using a Bio-Tek Synergy 2 Instrument (Winooski; VT).

### 2.7. Cellular Viability Measurement Using the Colorimetric MTT Reduction Assay

The tetrazolium salt dye is cleaved by live and metabolically active cells but not by dead cells; therefore, the MTT reduction assay was used to measure the viability of THP-1 cells treated with tunicamycin with or without LPS [41, 42]. Briefly, freshly grown THP-1 cells adjusted to 1 million cells/mL were treated with tunicamycin doses ranging from 0.5 to 10 *μ*g/mL and incubated overnight followed in some cases by 6 hr of exposure to LPS. Control cells treated with DMSO only were coincubated under the same experimental conditions. Five mg of thiazolyl blue tetrazolium bromide was dissolved in 1 mL of PBS and 15 *μ*L was added to 150 *μ*L of treated THP-1 cells which were further incubated at 37°C for one hour. Cells were centrifuged to remove extracellular dye and then lysed in 150 *μ*L of DMSO to dissolve the intracellular dye. The absorbance of reduced dye was measured at 591 nm wavelength using a Bio-Tek spectrophotometer.

### 2.8. Statistical Analysis

Mean values ± SD of three independent experiments, or two in few cases, were calculated with Microsoft Excel. Statistical analysis was performed with Prism v5.0 software using one-way ANOVA followed by Bonferroni multiple comparisons post hoc analysis. *P* values annotated ^***^ *P* < 0.0001; ^**^ *P* = 0.001–0.01; ^*^ *P* = 0.01–0.05; *P* > 0.05 were not significant.

## 3. Results

### 3.1. BDH2 Expression in Macrophages Is Downregulated by ER Stress and Inflammation

**Figure 1:**
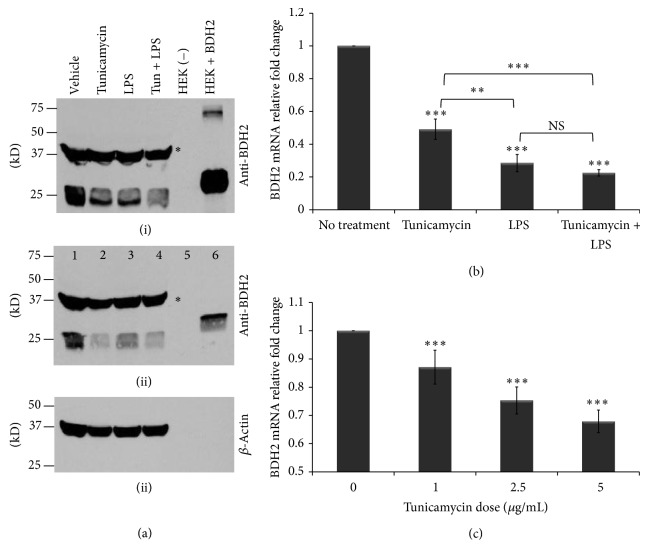
ER stress and inflammation downregulate BDH2 expression in macrophages. (a) Western blot analysis of BDH2 protein expression in THP-1 macrophage-like cells (1 × 10^6^ cells/mL) treated with tunicamycin (10 *μ*g/mL) or vehicle (DMSO) overnight prior to LPS exposure (40 ng/mL) for 6 hr. BDH2 protein was detected with monoclonal anti-BDH2 TrueMAB antibody clone 2G. Lane 1: vehicle-treated THP-1 cells; 2: tunicamycin treated; 3: LPS treated; 4: tunicamycin and LPS treated; 5: untransfected HEK293 cells as negative control; 6: BDH2 transfected HEK293 cells as positive control (BDH2 positive control contains a FLAG tag and 10-fold higher concentration was loaded in panel (i) compared to panel (ii), representing two independent experiments); ^*^nonspecific band. (b) THP-1 macrophages were treated with tunicamycin (10 *μ*g/mL) as above and RNA was extracted and BDH2 gene expression was assessed by quantitative RT-PCR normalized to that of *β*-actin. (c) THP-1 cells treated with tunicamycin doses 1, 2.5, or 5 *μ*g/mL and incubated overnight. The fold change in BDH2 gene expression was calculated in reference to DMSO treated control THP-1 cells using the ΔΔCT method and presented here normalized to vehicle (DMSO) treatment control. Error bars represent the SD from the mean of nine readouts generated from three independent experiments. ^*^ *P* values were calculated in reference to vehicle (DMSO) treatment control using one-way ANOVA followed by Bonferroni multiple comparisons post hoc analysis. *P* values annotated ^***^ *P* < 0.0001; ^**^ *P* = 0.001–0.01; *P* > 0.05 were not significant (NS).

**Figure 2:**
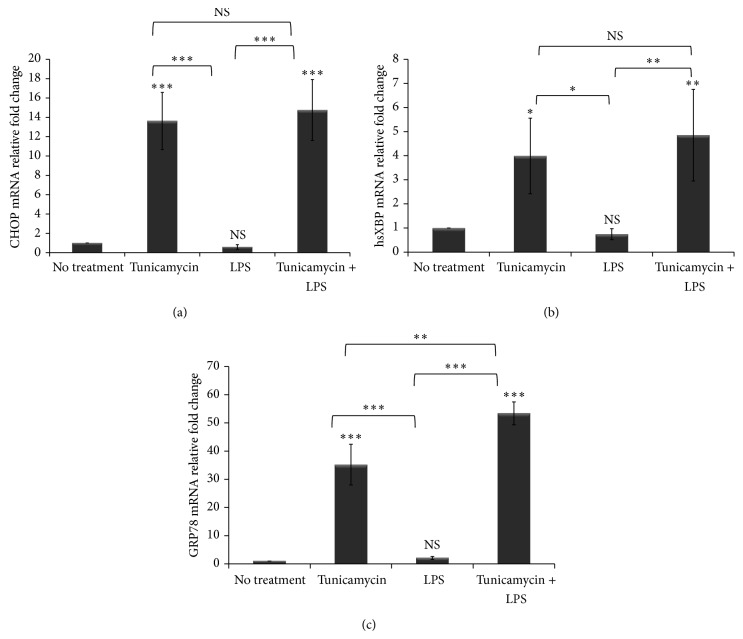
ER stress markers are induced in macrophages by tunicamycin. THP-1 cells (1 × 10^6^ cells/mL) were treated with tunicamycin (10 *μ*g/mL) or DMSO overnight prior to LPS exposure (40 ng/mL) for 6 hr. RNA was extracted and ER stress markers CHOP (a), hsXBP (b), and GRP78 (c) gene expressions were assessed by quantitative RT-PCR normalized to *β*-actin. The fold change in CHOP and hsXBP gene expression was calculated in reference to DMSO treated control THP-1 cells using the ΔΔCT method and presented here normalized to no treatment control. Error bars represent the SD from the mean of three independent experiments. ^*^ *P* values were calculated in reference to no treatment (DMSO only) control using one-way ANOVA followed by Bonferroni multiple comparisons post hoc analysis. *P* values annotated ^***^ *P* < 0.0001; ^**^ *P* = 0.001–0.01; ^*^ *P* = 0.01–0.05; *P* > 0.05 were not significant (NS).

Macrophages play a very important role in host defense and in iron homeostasis [1]. Recently, BDH2 was reported to play a crucial role in intracellular iron trafficking by catalyzing the synthesis of 2,5-DHBA [12]. Further, ER stress has been shown to dysregulate cellular iron homeostasis [30]. Zughaier et al. reported that BDH2 expression in macrophages is downregulated upon bacterial infection [15]. However, BDH2 expression and regulation in macrophages during ER stress and inflammation have not yet been described. To investigate BDH2 expression in macrophages and its regulation by ER stress and inflammation, we employed human macrophage-like monocytic cells (THP-1) treated with tunicamycin overnight prior to LPS exposure for 6 hr to induce ER stress and inflammation, respectively. Here we show that BDH2 protein and gene expression in macrophages was downregulated by ER stress and inflammation (Figure 1). Reduced BDH2 protein levels were observed in THP-1 cells after treatment with LPS and tunicamycin as compared to cells treated with DMSO alone (Figure 1(a)). Consistent with this observation, BDH2 gene expression was significantly downregulated when THP-1 macrophages were treated with tunicamycin and LPS either alone or in combination (Figure 1(b)). Treatment with tunicamycin alone reduced BDH2 mRNA expression in a dose-dependent manner (Figure 1(c)). Further, we examined the activation of ER stress markers and as expected overnight tunicamycin treatment significantly induced ER stress markers CHOP, hsXBP-1, GRP78 (Figure 2), and ATF6 (data not shown). In contrast, 6 hr LPS treatment alone did not upregulate ER stress markers in THP-1 cells (Figure 2), as documented previously [43]. Cellular viability was assessed using the MTT reduction assay [41] which showed that the doses of tunicamycin and LPS used here did not reduce cellular viability (see Supplementary Figure 1A in Supplementary Material available online at http://dx.doi.org/10.1155/2014/140728). These data indicate that ER stress and inflammation downregulate BDH2 expression in THP-1 macrophages.

### 3.2. Cellular Iron Homeostasis Is Dysregulated by ER Stress and Inflammation in Macrophages

**Figure 3:**
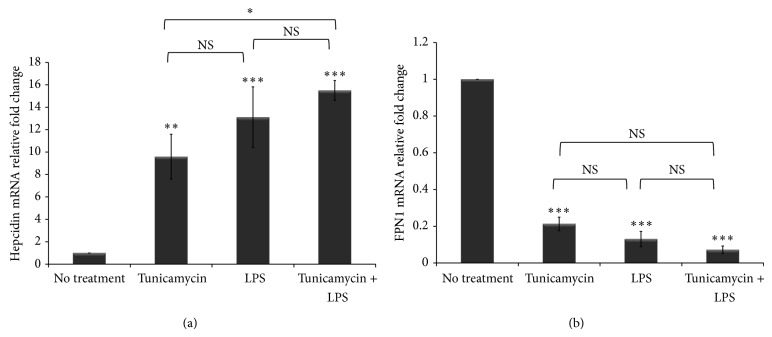
ER stress and inflammation dysregulate cellular iron homeostasis. THP-1 cells (1 × 10^6^ cells/mL) were treated with tunicamycin (10 *μ*g/mL) or DMSO overnight prior to LPS exposure (40 ng/mL) for 6 hr. ((a), (b)) Hepcidin and ferroportin (FPN1) gene expression was assessed by quantitative RT-PCR normalized to *β*-actin. The fold change in hepcidin and FPN1 gene expression was calculated in reference to DMSO treated control THP-1 cells using the ΔΔCT method and presented here normalized to no treatment control. Error bars represent the SD from the mean of three independent experiments. *P* values were calculated in reference to vehicle (DMSO) treatment control using one-way ANOVA followed by Bonferroni multiple comparisons post hoc analysis. *P* values annotated ^***^ *P* < 0.0001; ^**^ *P* = 0.001–0.01; ^*^ *P* = 0.01–0.05; *P* > 0.05 were not significant (NS).

**Figure 4:**
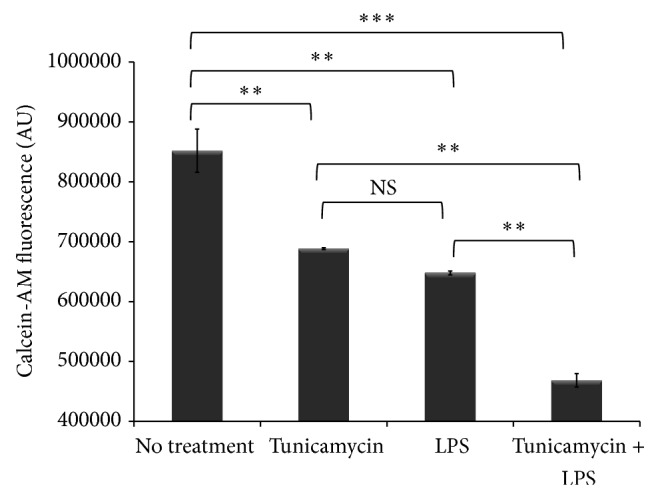
ER stress and inflammation induce iron retention in macrophages. THP-1 cells (1 × 10^6^ cells/mL) were treated with tunicamycin (10 *μ*g/mL) or DMSO overnight prior to LPS exposure (40 ng/mL) for 6 hr and iron retention in macrophages was determined using the calcein-AM fluorescent probe method [40]. DMSO treated cells were incubated simultaneously and used as controls. Calcein-AM fluorescence was measured by excitation at 488 nm and emission at 528 nm wavelength (see Section 2). Calcein-AM fluorescence is quenched upon binding iron and is therefore inversely correlated with intracellular iron accumulation. AU: arbitrary units. Error bars represent the SD from the mean of triplicate reading and data are representative of two independent experiments. *P* values were calculated in reference to vehicle (DMSO) treatment control using one-way ANOVA followed by Bonferroni multiple comparisons post hoc analysis. *P* values annotated ^***^ *P* < 0.0001; ^**^ *P* = 0.001–0.01; ^*^ *P* = 0.01–0.05; *P* > 0.05 were not significant (NS).

ER stress controls iron homeostasis via the induction of hepcidin, the master iron regulating hormone [2]. Hepcidin binds to the only known cellular iron exporter, ferroportin, and leads to its internalization and degradation, consequently inhibiting iron egress from macrophages [7]. The dysregulation of the hepcidin-ferroportin axis leads to iron retention in macrophages and increases the cytosolic labile iron pool which is highly reactive and causes oxidative stress [3]. Therefore, detoxifying labile iron is essential to maintaining cellular iron homeostasis. To investigate intracellular iron status in tunicamycin and LPS treated THP-1 macrophages we determined the expression of hepcidin and ferroportin. The data show that tunicamycin-induced ER stress as well as LPS-induced inflammation led to significant upregulation of hepcidin gene expression in THP-1 macrophages (Figure 3(a)). In contrast, tunicamycin and LPS treatment led to a dramatic reduction in ferroportin expression in macrophages (Figure 3(b)). Ferroportin allows iron egress from macrophages; therefore, its degradation leads to iron retention. We measured labile iron accumulation in macrophages using calcein-AM [40] and found that tunicamycin and LPS treatment led to significant iron retention (Figure 4 and Supplementary Figure 1B). A synergistic effect on iron retention was observed when macrophages were cotreated with tunicamycin and LPS. The results suggest that chemically induced ER stress and inflammation alter intracellular iron homeostasis in macrophages.

### 3.3. ER Stress and Inflammation Dramatically Induce NGAL Gene Expression

**Figure 5:**
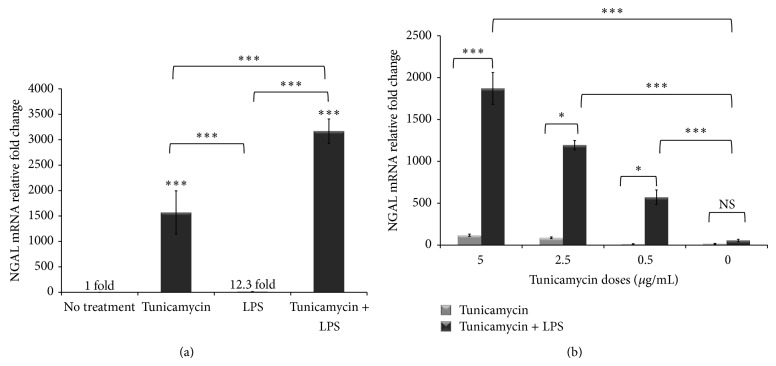
NGAL gene expression is massively upregulated by ER stress. (a) THP-1 cells (1 × 10^6^ cells/mL) were treated with tunicamycin (10 *μ*g/mL) or DMSO overnight prior to LPS exposure (40 ng/mL) for 6 hr. (b) THP-1 cells (1 × 10^6^ cells/mL) were treated with tunicamycin doses (5, 2.5, or 0.5 *μ*g/mL) as in panel (a) or tunicamycin followed by LPS (40 ng/mL). RNA was extracted and NGAL gene expression was assessed by quantitative RT-PCR normalized to *β*-actin. The fold change in NGAL gene expression was calculated in reference to DMSO treated control THP-1 cells using the ΔΔCT method and presented here normalized to vehicle (DMSO) treatment control. Error bars represent the SD from the mean of three independent experiments. ^*^ *P* values were calculated in reference to no treatment (DMSO only) control using one-way ANOVA followed by Bonferroni multiple comparisons post hoc analysis. *P* values annotated ^***^ *P* < 0.0001; ^**^ *P* = 0.001–0.01; ^*^ *P* = 0.01–0.05; *P* > 0.05 were not significant (NS).

NGAL plays a dual role in physiologic cellular iron homeostasis and in host defense. We recently reported that LPS induced NGAL secretion from peripheral monocytes [44]. ER stress is also reported to highly induce NGAL expression [45]. However, the impact of ER stress combined with inflammation on NGAL expression is not yet known. To this end, we determined NGAL gene expression in tunicamycin and LPS treated macrophages. As expected, LPS treatment led to significant upregulation of NGAL gene expression (~12-fold) but tunicamycin treatment led to a dramatic ~1500-fold increase in NGAL gene expression (Figure 5(a)). Tunicamycin induced NGAL gene expression in a dose-dependent manner (Figure 5(b)). A synergistic effect was observed when THP1 cells were cotreated with tunicamycin and LPS which resulted in an approximately ~3000-fold increase in NGAL gene expression (Figure 5(a)). This synergistic effect was tunicamycin dose-dependent (Figure 5(b)). Tunicamycin-induced ER stress led to ~100-fold greater increase in NGAL gene expression compared to LPS. The synergistic cross-talk between tunicamycin-induced ER stress and LPS-induced inflammation led to ~200-fold increase in NGAL gene expression compared to LPS alone. These data suggest that chemically induced ER stress dramatically increases NGAL expression more than inflammation but when combined a synergistic upregulation of NGAL is observed.

### 3.4. ER Stress and Inflammation Induce Heme Oxygenase-1 (HO-1) Gene Expression

**Figure 6:**
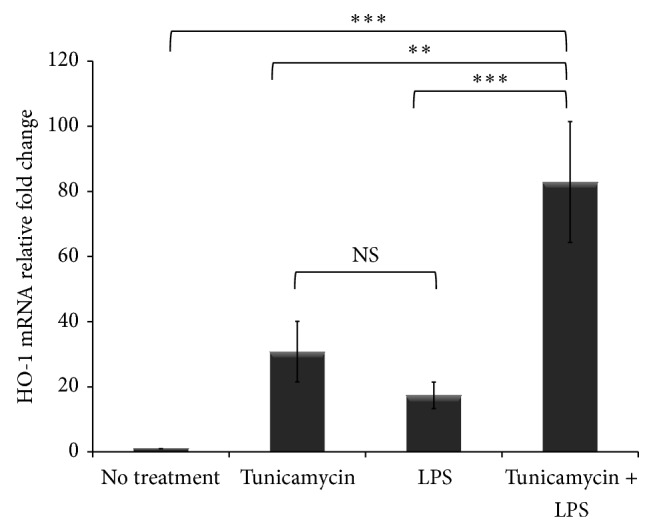
ER stress and inflammation induce heme oxygenase-1 (HO-1) gene expression in macrophages. THP-1 cells (1 × 10^6^ cells/mL) were treated with tunicamycin (10 *μ*g/mL) or DMSO overnight prior to LPS exposure (40 ng/mL) for 6 hr. RNA was extracted and HO-1 gene expression was assessed by quantitative RT-PCR normalized to *β*-actin. The fold change in OH-1 gene expression was calculated in reference to DMSO treated control THP-1 cells using the ΔΔCT method and presented here normalized to no treatment control. Error bars represent the SD from the mean of three independent experiments. *P* values were calculated in reference to vehicle treatment using one-way ANOVA followed by Bonferroni multiple comparisons post hoc analysis. *P* values annotated ^***^ *P* < 0.0001; ^**^ *P* = 0.001–0.01; *P* > 0.05 were not significant (NS).

HO-1 is a stress-induced anti-inflammatory [19, 21, 22] cytoprotective enzyme that catalyzes the degradation of heme and consequently liberates iron and carbon monoxide [20]. The released carbon monoxide suppresses hepcidin expression; therefore, HO-1 plays a role in regulating iron homeostasis [46]. Our data demonstrate that ER stress and inflammation dysregulate intracellular iron homeostasis in macrophages. Therefore, we investigated the impact of ER stress and inflammation on HO-1 expression in macrophages as an anti-inflammatory immune response. Here we show that HO-1 gene expression is significantly upregulated in THP-1 cells treated with tunicamycin or LPS alone (Figure 6). However, the combination of tunicamycin and LPS treatment synergistically upregulated HO-1 expression in THP-1 cells (Figure 6) suggesting a potent response to combat cellular stress.

### 3.5. Vitamin D Restores BDH2 Expression in LPS-Stimulated Macrophages

**Figure 7:**
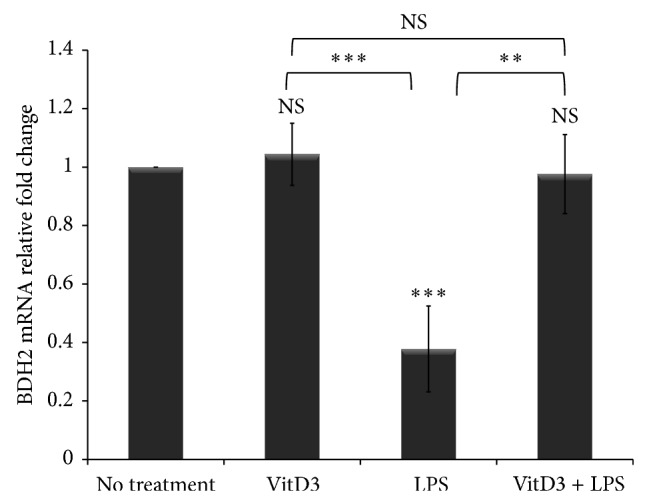
Vitamin D pretreatment protects BDH2 expression in LPS-stimulated macrophages. THP-1 macrophage-like cells (1 × 10^6^ cells/mL) were treated with 20 nM of 1,25(OH)_2_D_3_, the hormonally active form of vitamin D_3_, overnight prior to LPS exposure (40 ng/mL) for 6 hr. RNA was extracted and BDH2 gene expression was assessed by quantitative RT-PCR normalized to *β*-actin. The fold change in BDH2 gene expression was calculated in reference to DMSO treated control THP-1 cells using the ΔΔCT method and presented here normalized to no treatment control. VitD_3_: 1,2(OH)_2_D_3_. Error bars represent the SD from the mean of three independent experiments. *P* values were calculated in reference to vehicle treatment using one-way ANOVA followed by Bonferroni multiple comparisons post hoc analysis. *P* values annotated ^***^ *P* < 0.0001; ^**^ *P* = 0.001–0.01; *P* > 0.05 were not significant (NS).

In this report we show that tunicamycin-induced ER stress and LPS-induced inflammation downregulate BDH2 expression in macrophages and alter intracellular iron homeostasis. Vitamin D possesses immune modulatory activity and exerts an anti-inflammatory effect [47–49]. Zughaier et al. found that vitamin D plays an important role in regulating the iron-hepcidin-ferroportin axis in monocytes by restoring hepcidin and ferroportin gene expression and reducing IL-6 and IL-1*β* cytokine release [50]. Vitamin D was also reported to suppress ER stress in macrophages and therefore is a natural ER stress reliever [51, 52]. We hypothesized that vitamin D may restore BDH2 expression in LPS stimulated macrophages. To test our hypothesis, THP-1 macrophages were treated with 20 nM of 1,25 dihydroxy vitamin D_3_ (1,25(OH)_2_D_3_), the hormonally active form of vitamin D_3_, for 24 hr prior to LPS exposure. Here we report for the first time that vitamin D_3_ pretreatment protects macrophages from the LPS-induced reduction in BDH2 gene expression (Figure 7). Treatment with vitamin D_3_ alone did not significantly induce BDH2 expression but LPS treatment led to significant downregulation of BDH2 expression which was absent in cells pretreated with vitamin D_3_ prior to LPS. Taken together, the data suggest that downregulation of BDH2 gene expression by LPS, which is a common model of inflammation, can be restored by vitamin D_3_.

### 3.6. ER Stress Dysregulates Cytokine and Chemokine Release from LPS-Stimulated Macrophages

**Figure 8:**
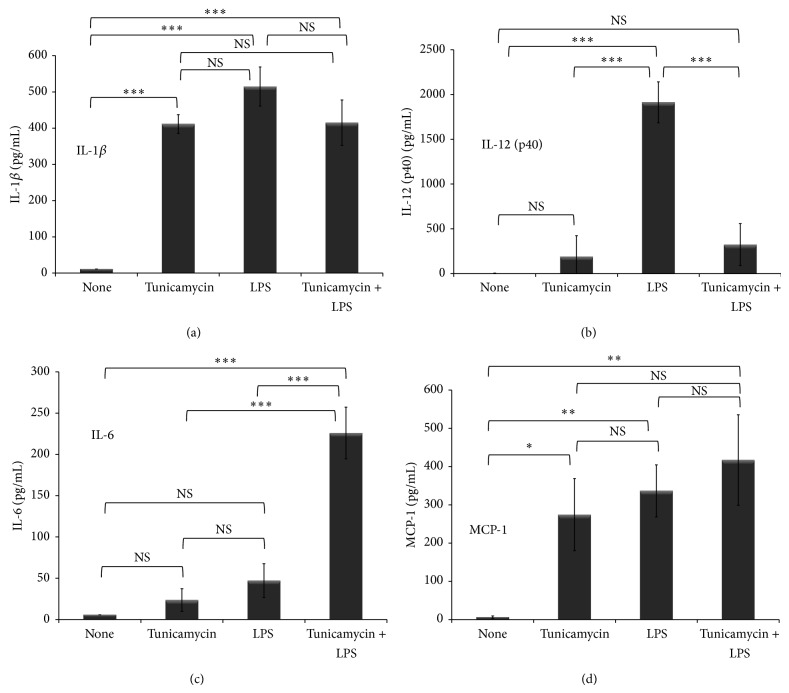
Induction of ER stress dysregulates LPS-induced inflammatory cytokine release. THP-1 cells (1 × 10^6^ cells/mL) were treated with tunicamycin (10 *μ*g/mL) or DMSO overnight prior to LPS exposure (40 ng/mL) for 6 hr. Supernatants were collected and 50 *μ*L was used to measure cytokine and chemokine release using the Luminex based magnetic bead assay (see Section 2). A selected panel of cytokine and chemokine release is presented. (a) IL-1*β*, (b) IL-12p40, (c) IL-6, and (d) MCP-1. Error bars represent the SD from the mean of three independent experiments. *P* values annotated ^***^ *P* < 0.0001; ^**^ *P* = 0.001–0.01; ^*^ *P* = 0.01–0.05; *P* > 0.05 were not significant (NS).

Upon exposure to LPS, macrophages respond by releasing large quantities of inflammatory mediators, an innate immune response known as the cytokine storm. Dysregulated levels of inflammatory mediators contribute to dysfunctional immune responses. During ER stress, macrophages downregulate* de novo* protein synthesis as an adaptation process to restore ER homeostasis, which results in reduced secretion of some inflammatory mediators [53, 54]. In this report we show that ER stress combined with inflammation dysregulated macrophage iron homeostasis. Therefore, we examined the impact of ER stress combined with inflammation on release of inflammatory mediators from macrophages. Using Luminex technology, we evaluated cytokine and chemokine release from cells treated with tunicamycin (10 *μ*g/mL) to induce ER stress prior to LPS (40 ng/mL) exposure for 6 hr to induce the cytokine storm. As expected, LPS exposure without tunicamycin treatment induced massive release of cytokines (IL-1*β*, TNF*α*, IL-6, IL-12p40, IFN*α*, IL-1R antagonist, and IL-2R) and chemokines (RANTES, MCP-1, MIP1*α*, MIP1*β*, IP-10/CXCL10, and IL-8) compared to unstimulated THP-1 macrophages (Table 1). Tunicamycin treatment alone (without LPS exposure) also induced cytokine/chemokine release which was typically ~2- to 5-fold less than LPS treatment (Figures 8(a), 8(b), 8(c), and 8(d) and Table 1). Since ER stress downregulates protein synthesis machinery [55], tunicamycin treatment followed by LPS exposure led to significant reduction in cytokines (IL-12p40, IL-1R antagonist) and chemokines (MIP1*α*, MIP1*β*) compared to LPS exposure alone. A slight reduction was observed in other cytokines and chemokines released from macrophages exposed to both tunicamycin and LPS (Table 1). In contrast, a significant and synergistic increase in IL-6 release was observed when THP-1 macrophages were exposed to both tunicamycin and LPS. IL-6 is a proinflammatory cytokine that plays a significant role in systemic iron metabolism by directly inducing the expression of hepcidin, the master iron-regulating hormone [8]. Therefore, tunicamycin-induced ER stress combined with inflammation upregulates IL-6, consequently contributing to altered iron homeostasis.

## 4. Discussion

Limiting the bioavailability of iron during infection is a host defense mechanism driven by the hepcidin-ferroportin axis [2, 7, 15] that leads to iron retention in macrophages. In this report we describe the downregulation of BDH2 in macrophages in response to ER stress and inflammation. We show that chemically induced ER stress combined with LPS-induced inflammation downregulates BDH2 expression in human THP-1 macrophages. The physiological role of BDH2 in intracellular iron homeostasis was described recently [12]. BDH2 catalyzes the synthesis of the mammalian siderophore 2,5DHBA that protects from oxidant stress by capturing free toxic iron. Cells lacking a functional siderophore retain high levels of free iron leading to increased ROS production and iron-deficient mitochondria [12]. More recently, Liu and coworkers also reported that BDH2 contains an iron-responsive element and thereby is regulated by intracellular iron levels [14]. Zughaier et al. reported the downregulation of BDH2 gene expression in macrophages in response to bacterial infection [15]. Here we report a synergistic effect of ER stress combined with inflammation on the downregulation of BDH2 gene and protein expression in macrophages which led to dysregulated cytokine release and iron retention. Our finding is novel and sheds light on the role of BDH2 in iron-limiting innate immunity.

Iron homeostasis directly influences macrophage function [56]. Recent evidence showed that iron retention dictates macrophage polarization [5, 57] and induces unrestrained proinflammatory macrophages that impair wound healing [58]. ER stress induces hepcidin via CHOP and CREB [30]. During infection, hepcidin is directly induced by LPS via TLR4 activation [59] and during inflammation hepcidin is induced by IL-6 [8]. Elevated hepcidin expression leads to the development of hypoferremia or anemia of chronic inflammation [2]. Our data show that chemically induced ER stress and LPS-induced inflammation dysregulated the hepcidin-ferroportin axis and led to iron retention in THP-1 macrophages and consequently downregulated BDH2 expression. Devireddy and coworkers showed that downregulating BDH2 expression using siRNA resulted in the depletion of the mammalian siderophore which caused iron retention in a murine pro-B lymphocytic cell line, reduced mitochondrial iron, and consequently reduced heme synthesis [12].

**Figure 9:**
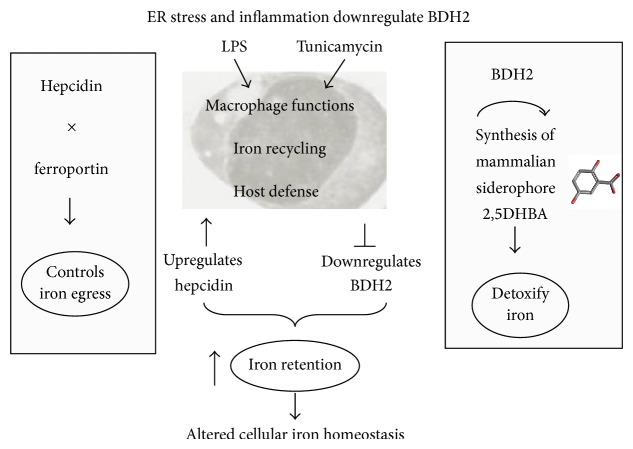
Schematic presentation of BDH2 downregulation in macrophages. BDH2 is the rate-limiting enzyme in the synthesis of the mammalian siderophore 2,5DHBA that detoxifies and traffics intracellular labile iron. Treatment of THP-1 macrophage-like monocytes with LPS or tunicamycin downregulates BDH2 expression leading to intracellular labile iron accumulation. Hepcidin is the master iron regulating hormone that binds to the only iron exporter ferroportin causing its internalization and degradation consequently preventing iron egress from macrophages. LPS and tunicamycin treatment increases hepcidin expression and downregulates ferroportin causing iron retention in macrophages. The downregulation of BDH2 contributes to altered cellular iron homeostasis.

BDH2 is a homolog of the bacterial enzyme EntA that catalyzes the synthesis of the bacterial siderophore, enterobactin [60]. NGAL sequesters enterobactin thereby limiting bacterial growth and traffics iron-bound complexes across tissues. Thus, in addition to their physiological roles in iron homeostasis, NGAL and BDH2 constitute the iron-limiting arm of innate immune defense. Further, NGAL is highly induced as an antioxidant response during oxidative stress [61] and in response to ER stress [45]. Our data demonstrate a synergistic effect of ER stress and inflammation on the dramatic upregulation of NGAL. Elevated levels of NGAL are associated with many chronic diseases like kidney disease, heart disease, arthritis, chronic obstructive pulmonary disease, and cancer [62–64]. High levels of NGAL were shown to deactivate macrophages and worsen outcomes of pneumococcal pneumonia [65]. We recently reported that circulating NGAL levels are elevated in subjects with cystic fibrosis compared to healthy donors and demonstrated that peripheral monocytes secrete NGAL in response to LPS and to* Pseudomonas aeruginosa* infection [44]. Moreover, NGAL expression is upregulated whereas BDH2 expression is downregulated in macrophages upon bacterial infection with* Neisseria gonorrhoeae* [15]. Altered iron homeostasis manifesting as hypoferremia is common across all these diseases. Taken together, ER stress combined with inflammation downregulates BDH2 expression consequent to the dysregulation of hepcidin-ferroportin axis leading to iron retention in macrophages and dramatic NGAL upregulation which contribute to altered intracellular iron homeostasis (Figure 9).

ER stress has been shown to induce chronic inflammation in metabolic and chronic heart diseases [66, 67]. Tunicamycin, which inhibits protein glycosylation and triggers the unfolded protein response [68], combined with LPS synergistically increased IL-6 release from macrophages. Hepcidin expression is directly induced by IL-6. Previous study showed that treatment with anti-IL-6 antibody lowered hepcidin levels which alleviated hypoferremia and restored iron homeostasis [2, 69]. Among many inflammatory mediators IL-6 is a predictor of mortality in chronic kidney disease [70], in HIV [71], and in other chronic diseases associated with immune activation due to microbial translocation [72, 73]. Our results suggest that ER stress combined with inflammation increases IL-6 that induces hepcidin expression leading to increased intracellular iron which downregulates BDH2 expression. Further, NGAL possesses an immunomodulatory activity and exerts a suppressive effect on macrophages leading to reduced cytokine release [65, 74]. We observed a synergistic effect of ER stress combined with inflammation on the dramatic upregulation of NGAL expression which may contribute to dysregulated cytokine and chemokine response in macrophages. In this report we describe a mechanism by which ER stress combined with inflammation contributes to dysfunctional iron-limiting innate immune responses in macrophages.

Vitamin D possesses immunomodulatory activities and lowers circulating cytokines* in vivo* [47, 48, 75]. Recent study demonstrated that vitamin D relieves ER stress in macrophages [52]. We reported that the hormonally active form of vitamin D, 1,25 dihydroxyvitamin D_3_, reduced MCP-1 release from macrophages treated with LPS or infected with* Pseudomonas aeruginosa* bacteria [47]. More recently, Zughaier et al. showed that 1,25 dihydroxyvitamin D_3_ treatment reduced IL-6 release from LPS activated macrophages and reduced hepcidin gene expression while upregulating ferroportin gene expression in a dose-dependent manner; thereby, vitamin D restored the hepcidin-ferroportin axis in macrophages [50]. Taken together, our data show that ER stress and inflammation downregulate BDH2 expression and vitamin D treatment restored BDH2 expression in LPS activated macrophages. This is the first report to show that vitamin D restores BDH2 expression. It is not known whether vitamin D exerts direct or indirect regulatory effects on BDH2 gene expression but it is worthy of further investigation.

**Figure 10:**
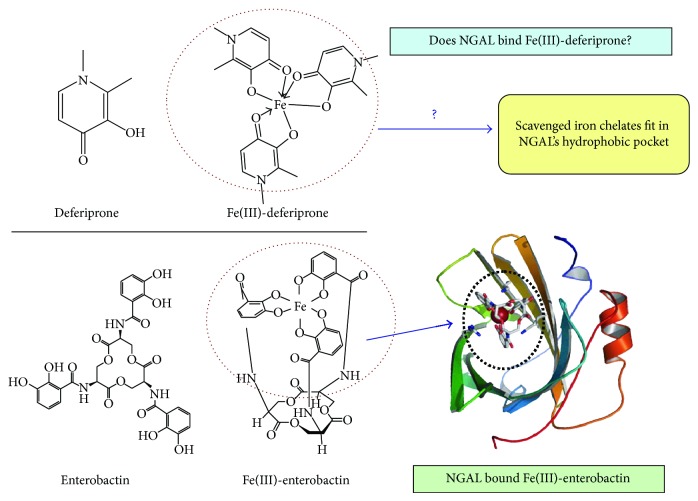
A schematic presentation of proposed small molecule iron chelator acting similar to the mammalian siderophore 2,5-dihydroxybenzoic acid. The DFP-iron complex shares structural resemblance to enterobactin, the major bacterial siderophore that binds iron with very high affinity. As a defense mechanism, the host upregulates NGAL expression to scavenge enterobactin which in turn limits iron availability and inhibits bacterial growth. DFP is an iron chelating small molecule drug that gains access to cells and scavenges free intracellular iron and then exits as a DFP-iron complex. Based on the structural resemblance to enterobactin-iron complexes, the DFP-iron complex is expected to bind NGAL by fitting into the hydrophobic pocket [79]. Therefore, small molecule iron chelators may act as the mammalian siderophore and redistribute iron retained in macrophages.

The important role of BDH2 in intracellular homeostasis relies on the production of the mammalian siderophore 2,5-DHBA that binds free iron and thereby restores cellular iron homeostasis [17]. We propose that the synthetic iron chelating drug Deferiprone (DFP: 3-hydroxy-1,2-dimethylpyridin-4(1*H*)-one) [76, 77], commonly used to treat iron overload disorders [78], may emulate the mammalian siderophore and redistribute iron retained in macrophages. DFP is a small molecule that diffuses across the cellular membrane and scavenges free intracellular iron. Three molecules of DFP make a complex with one ferric iron molecule [76]. This DFP-iron complex shares structural resemblance to enterobactin, the major bacterial siderophore that binds iron with very high affinity (Figure 10). In response to infection and as a defense mechanism, the host upregulates NGAL expression to scavenge enterobactin which in turn limits iron availability and inhibits bacterial growth. Our data show that NGAL is dramatically upregulated in response to ER stress and inflammation. We hypothesize that, upon downregulation of BDH2 expression in macrophages, DFP may compensate for the reduction in mammalian siderophore production. DFP gains access to cells and scavenges free intracellular iron and then exits as a DFP-iron complex. Based on the structural resemblance to enterobactin-iron complexes, the DFP-iron complex is expected to bind NGAL by fitting into the hydrophobic pocket [79] (Figure 10). NGAL-siderophore complexes are then excreted to urine. Therefore, DFP could be a potential therapeutic for altered cellular iron homeostasis that lowers both intracellular iron and abnormal NGAL levels, consequently restoring intracellular iron homeostasis in macrophages. Our hypothesis is under current investigation.

## 5. Conclusion

Inflammation combined with ER stress led to massive BDH2 downregulation and increased the expression of ER stress markers, upregulated hepcidin expression, downregulated ferroportin expression, caused iron retention in macrophages, and dysregulated cytokine release from macrophages. ER stress combined with inflammation synergistically upregulated the expression of the iron carrier protein NGAL and the stress-inducible heme degrading enzyme heme oxygenase-1 (HO-1) leading to iron liberation. However, vitamin D treatment prior to LPS exposure restored BDH2 expression. BDH2 is expressed in many types of cells [80]; therefore, downregulation of BDH2 may have detrimental effects on cellular functions. Downregulation of BDH2 during infection is due to activation of hepcidin and intracellular iron retention, indicating an important role for BDH2 in iron-limiting innate immunity and host defense. Our data suggest that continued ER stress combined with inflammation impacts on BDH2 expression and contributes to altered intracellular iron homeostasis.

## Supplementary Material

Supplementary Figure 1: Cellular viability and iron retention in tunicamycin treated macrophages. A: Cellular viability of THP-1 cells treated with tunicamycin doses prior to LPS exposure was assessed using the MTT assay (MTT OD-A591). B: THP-1 cells (1x10^6^ cell/ml) were treated with tunicamycin doses or DMSO overnight prior to LPS exposure (40 ng/ml) for 6 hr and iron retention in macrophages was determined using calcein-AM. DMSO treated cells were incubated simultaneously and used as controls. Calcein-AM fluorescence is quenched upon binding iron and is therefore inversely correlated with intracellular iron accumulation. AU: arbitrary units. Error bars represent the SD from the mean of triplicate readings and data are representative of two independent experiments.

## Figures and Tables

**Table 1:** Cytokine and chemokine release from THP-1 cells.

Cytokine/chemokine release (pg/mL)	No treatment	Tunicamycin (10 *µ*g/mL)^a,b^	LPS (40 ng/mL)^a,b,c,#^	Tunicamycin + LPS^a,c^
IL-1*β*	11.4 ± 0.1	411.6 ± 25.7	515 ± 54	415.3 ± 62
*P* value < 0.0001^a^		NS^b^		NS^c^

IL-13	13.1 ± 1.3	18 ± 2.5	19.43 ± 2.5	21.24 ± 2.2
*P* value = 0.0142^a^		NS^b^		NS^c^

IL-6	5.69 ± 0.1	23.6 ± 13.7	47.2 ± 20	225 ± 31
*P* value < 0.0001^a^		NS^b^		<0.0001^c^

IL-12p40	2.9 ± 2.1	190 ± 232	1913 ± 228	323 ± 234
*P* value < 0.0001^a^		<0.0001^b^		<0.0001^c^

GM-CSF	ND^!^	2.02 ± 0.7	2.98 ± 0.39	3.7 ± 0.38
*P* value < 0.0001^a^		NS^b^		NS^c^

IFN*α*	7.81 ± 1.4	21.8 ± 6.2	35.9 ± 0.73	28.8 ± 4.6
*P* value = 0.0001^a^		<0.01^b^		NS^c^

IL-1R antagonist	11.33 ± 6.6	430.9 ± 114	1071 ± 164	528 ± 41.2
*P* value < 0.0001^a^		<0.0001^b^		<0.001^c^

TNF*α*	0.714 ± 0.15	40.2 ± 58	245.2 ± 152	30.8 ± 38
*P* value = 0.025^a^		NS^b^		NS^c^

IL-2R	2.27 ± 3.0	47.9 ± 35.8	122.8 ± 7.7	102.7 ± 10.4
*P* value = 0.0002^a^		<0.001^b^		NS^c^

RANTES	67 ± 48.7	373 ± 169	756 ± 82	480 ± 112
*P* value = 0.0005^a^		<0.01^b^		NS^c^

MIP1*α*	34.29 ± 0	1010 ± 1493	5404 ± 394	2279 ± 397
*P* value = 0.001^a^		<0.001^b^		<0.01^c^

MIP1*β*	9.8 ± 0	1913 ± 1750	8295 ± 1895	3902 ± 551
*P* value = 0.0003^a^		<0.001^b^		<0.01^c^

MCP-1	5.09 ± 2.2	274 ± 94	336 ± 68	417 ± 118
*P* value = 0.0015^a^		NS^b^		NS^c^

IP-10 (CXCL10)	4.0 ± 3.5	67.5 ± 56	104.4 ± 67	55.1 ± 29
*P* value = 0.139^a^		NS^b^		NS^c^

IL-8	15.4 ± 5.7	6832 ± 542	7034 ± 848	6832 ± 598
*P* value < 0.0001^a^		NS^b^		NS^c^

^!^ND: not detected.

^a^ *P* values calculated in reference to no treatment using one-way ANOVA.

^b^ *P* values of LPS treatment (^#^) compared to tunicamycin using Bonferroni's multiple comparison test.

^c^ *P* values of LPS treatment (^#^) compared to tunicamycin + LPS using Bonferroni's multiple comparison test.

NS: not significant.

## Abbreviations

ER stress: Endoplasmic reticulum stress
LPS: Lipopolysaccharide
BDH2: Butyrate dehydrogenase type 2
FPN1: Ferroportin
NGAL: Neutrophil gelatinase-associated lipocalin
UPR: Unfolded protein response
CHOP: Transcription factor C/EBP homolog protein
HsXBP-1: X-box binding protein 1, spiced isoform
GRP78: Glucose regulating protein 78
ATF6: Activating transcription factor 6
MTT: Thiazolyl blue tetrazolium bromide
DHBA: Dihydroxybenzoic acid
ROS: Reactive oxygen species
DMSO: Dimethylsulfoxide
IL-1*β*: Interleukin 1 beta
TNF*α*: Tumor necrosis factor alpha
IL-13: Interleukin 13
IL-6: Interleukin 6
IL-12(p40): Interleukin 12 subunit 40
GM-CSF: Granulocyte macrophage colony-stimulating factor
INF*α*: Interferon alpha
IL-1R antagonist: Interleukin 1 receptor antagonist
IL-2R: Interleukin 2 receptor
MIP1*α*: Macrophage inflammatory protein 1 alpha
MIP1*β*: Macrophage inflammatory protein 1 beta
MCP-1: Monocyte chemotactic protein
IP-10: Inducible small protein 10
IL-8: Interleukin 8.

## References

[1] Wang J., Pantopoulos K. (2011). Regulation of cellular iron metabolism. *Biochemical Journal*.

[2] Ganz T., Nemeth E. (2011). Hepcidin and disorders of iron metabolism. *Annual Review of Medicine*.

[3] Lipinski P., Starzynski R. R., Stys A., Stracilo M. (2010). Iron homeostasis, a defense mechanism in oxidative stress. *Postepy Biochemii*.

[4] Pan X., Tamilselvam B., Hansen E. J., Daefler S. (2010). Modulation of iron homeostasis in macrophages by bacterial intracellular pathogens. *BMC Microbiology*.

[5] Recalcati S., Locati M., Marini A., Santambrogio P., Zaninotto F., De Pizzol M., Zammataro L., Girelli D., Cairo G. (2010). Differential regulation of iron homeostasis during human macrophage polarized activation. *European Journal of Immunology*.

[6] Ganz T. (2002). The role of hepcidin in iron sequestration during infections and in the pathogenesis of anemia of chronic disease. *The Israel Medical Association Journal: IMAJ*.

[7] Nemeth E., Tuttle M. S., Powelson J., Vaughn M. D., Donovan A., Ward D. M., Ganz T., Kaplan J. (2004). Hepcidin regulates cellular iron efflux by binding to ferroportin and inducing its internalization. *Science*.

[8] Nemeth E., Rivera S., Gabayan V., Keller C., Taudorf S., Pedersen B. K., Ganz T. (2004). IL-6 mediates hypoferremia of inflammation by inducing the synthesis of the iron regulatory hormone hepcidin. *The Journal of Clinical Investigation*.

[9] Nemeth E., Valore E. V., Territo M., Schiller G., Lichtenstein A., Ganz T. (2003). Hepcidin, a putative mediator of anemia of inflammation, is a type II acute-phase protein. *Blood*.

[10] Theurl M., Theurl I., Hochegger K., Obrist P., Subramaniam N., Van Rooijen N., Schuemann K., Weiss G. (2008). Kupffer cells modulate iron homeostasis in mice via regulation of hepcidin expression. *Journal of Molecular Medicine*.

[11] Theurl I., Theurl M., Seifert M., Mair S., Nairz M., Rumpold H., Zoller H., Bellmann-Weiler R., Niederegger H., Talasz H., Weiss G. (2008). Autocrine formation of hepcidin induces iron retention in human monocytes. *Blood*.

[12] Devireddy L. R., Hart D. O., Goetz D. H., Green M. R. (2010). A mammalian siderophore synthesized by an enzyme with a bacterial homolog involved in enterobactin production. *Cell*.

[13] Guo K., Lukacik P., Papagrigoriou E. (2006). Characterization of human DHRS6, an orphan short chain dehydrogenase/reductase enzyme: a novel, cytosolic type 2 R-*β*-hydroxybutyrate dehydrogenase. *The Journal of Biological Chemistry*.

[14] Liu Z., Lanford R., Mueller S., Gerhard G. S., Luscieti S., Sanchez M., Devireddy L. (2012). Siderophore-mediated iron trafficking in humans is regulated by iron. *Journal of Molecular Medicine*.

[15] Zughaier S. M., Kandler J. L., Shafer W. M. (2014). Neisseria gonorrhoeae modulates iron-limiting innate immune defenses in macrophages. *PLoS ONE*.

[16] Liu Z., Reba S., Chen W.-D., Porwal S. K., Boom W. H., Petersen R. B., Rojas R., Viswanathan R., Devireddy L. (2014). Regulation of mammalian siderophore 2,5-DHBA in the innate immune response to infection. *Journal of Experimental Medicine*.

[17] Liu Z., Ciocea A., Devireddy L. (2014). Endogenous siderophore 2,5-dihydroxybenzoic acid deficiency promotes anemia and splenic iron overload in mice. *Molecular and Cellular Biology*.

[18] Liu Z., Velpula K. K., Devireddy L. (2014). 3-Hydroxybutyrate dehydrogenase-2 and ferritin-H synergistically regulate intracellular iron. *The FEBS Journal*.

[19] Agarwal A., Bolisetty S. (2013). Adaptive responses to tissue injury: role of heme oxygenase-1. *Transactions of the American Clinical and Climatological Association*.

[20] Hull T. D., Bolisetty S., Dealmeida A. C., Litovsky S. H., Prabhu S. D., Agarwal A., George J. F. (2013). Heme oxygenase-1 expression protects the heart from acute injury caused by inducible Cre recombinase. *Laboratory Investigation*.

[21] Mandal P., Roychowdhury S., Park P.-H., Pratt B. T., Roger T., Nagy L. E. (2010). Adiponectin and heme oxygenase-1 suppress TLR4/MyD88-independent signaling in rat Kupffer cells and in mice after chronic ethanol exposure. *The Journal of Immunology*.

[22] Mandal P., Pritchard M. T., Nagy L. E. (2010). Anti-inflammatory pathways and alcoholic liver disease: role of an adiponectin/interleukin-10/heme oxygenase-1 pathway. *World Journal of Gastroenterology*.

[23] Packiam M., Wu H., Veit S. J., Mavrogiorgos N., Jerse A. E., Ingalls R. R. (2012). Protective role of Toll-like receptor 4 in experimental gonococcal infection of female mice. *Mucosal Immunology*.

[24] Mishra J., Dent C., Tarabishi R., Mitsnefes M. M., Ma Q., Kelly C., Ruff S. M., Zahedi K., Shao M., Bean J., Mori K., Barasch J., Devarajan P. (2005). Neutrophil gelatinase-associated lipocalin (NGAL) as a biomarker for acute renal injury after cardiac surgery. *The Lancet*.

[25] Chakraborty S., Kaur S., Guha S., Batra S. K. (2012). The multifaceted roles of neutrophil gelatinase associated lipocalin (NGAL) in inflammation and cancer. *Biochimica et Biophysica Acta: Reviews on Cancer*.

[26] Bao G., Clifton M., Hoette T. M., Mori K., Deng S.-X., Qiu A., Viltard M., Williams D., Paragas N., Leete T., Kulkarni R., Li X., Lee B., Kalandadze A., Ratner A. J., Pizarro J. C., Schmidt-Ott K. M., Landry D. W., Raymond K. N., Strong R. K., Barasch J. (2010). Iron traffics in circulation bound to a siderocalin (Ngal)—catechol complex. *Nature Chemical Biology*.

[27] Goetz D. H., Holmes M. A., Borregaard N., Bluhm M. E., Raymond K. N., Strong R. K. (2002). The neutrophil lipocalin NGAL is a bacteriostatic agent that interferes with siderophore-mediated iron acquisition. *Molecular Cell*.

[28] Flo T. H., Smith K. D., Sato S., Rodriguez D. J., Holmes M. A., Strong R. K., Akira S., Aderem A. (2004). Lipocalin 2 mediates an innate immune response to bacterial infection by sequestrating iron. *Nature*.

[29] Peek M. E., Bhatnagar A., McCarty N. A., Zughaier S. M. (2012). Pyoverdine, the Major Siderophore in *Pseudomonas aeruginosa*, Evades NGAL Recognition. *Interdisciplinary Perspectives on Infectious Diseases*.

[30] de Sousa M., Oliveira S. J., Pinto J. P. (2011). ER stress and iron homeostasis: a new frontier for the UPR. *Biochemistry Research International*.

[31] Yin J.-J., Li Y.-B., Wang Y., Liu G.-D., Wang J., Zhu X.-O., Pan S.-H. (2012). The role of autophagy in endoplasmic reticulum stress-induced pancreatic *β* cell death. *Autophagy*.

[32] Suh D. H., Kim M.-K., Kim H. S., Chung H. H., Song Y. S. (2012). Unfolded protein response to autophagy as a promising druggable target for anticancer therapy. *Annals of the New York Academy of Sciences*.

[33] Vecchi C., Montosi G., Zhang K., Lamberti I., Duncan S. A., Kaufman R. J., Pietrangelo A. (2009). ER stress controls iron metabolism through induction of hepcidin. *Science*.

[34] Oliveira S. J., Pinto J. P., Picarote G., Costa V. M., Carvalho F., Rangel M., de Sousa M., de Almeida S. F. (2009). ER stress-inducible factor CHOP affects the expression of hepcidin by modulating C/EBPalpha activity. *PLoS ONE*.

[35] Vidal R., Caballero B., Couve A., Hetz C. (2011). Converging pathways in the occurrence of endoplasmic reticulum (ER) stress in Huntington's disease. *Current Molecular Medicine*.

[36] Matus S., Glimcher L. H., Hetz C. (2011). Protein folding stress in neurodegenerative diseases: a glimpse into the ER. *Current Opinion in Cell Biology*.

[37] Qu X., Zou Z., Sun Q., Luby-Phelps K., Cheng P., Hogan R. N., Gilpin C., Levine B. (2007). Autophagy gene-dependent clearance of apoptotic cells during embryonic development. *Cell*.

[38] Zughaier S. M., Tzeng Y.-L., Zimmer S. M., Datta A., Carlson R. W., Stephens D. S. (2004). *Neisseria meningitidis* lipooligosaccharide structure-dependent activation of the macrophage CD14/Toll-like receptor 4 pathway. *Infection and Immunity*.

[39] Zughaier S. M. (2011). Neisseria meningitidis capsular polysaccharides induce inflammatory responses via TLR2 and TLR4-MD-2. *Journal of Leukocyte Biology*.

[40] Kakhlon O., Cabantchik Z. I. (2002). The labile iron pool: characterization, measurement, and participation in cellular processes. *Free Radical Biology & Medicine*.

[41] Gerlier D., Thomasset N. (1986). Use of MTT colorimetric assay to measure cell activation. *Journal of Immunological Methods*.

[42] Mosmann T. (1983). Rapid colorimetric assay for cellular growth and survival: application to proliferation and cytotoxicity assays. *Journal of Immunological Methods*.

[43] Nakayama Y., Endo M., Tsukano H., Mori M., Oike Y., Gotoh T. (2010). Molecular mechanisms of the LPS-induced non-apoptotic ER stress-CHOP pathway. *The Journal of Biochemistry*.

[44] Zughaier S. M., Tangpricha V., Leong T., Stecenko A. A., McCarty N. A. (2013). Peripheral monocytes derived from patients with cystic fibrosis and healthy donors secrete ngal in response to *Pseudomonas aeruginosa* infection. *Journal of Investigative Medicine*.

[45] Mahadevan N. R., Rodvold J., Almanza G., Pérez A. F., Wheeler M. C., Zanetti M. (2011). ER stress drives Lipocalin 2 upregulation in prostate cancer cells in an NF-*κ*B-dependent manner. *BMC Cancer*.

[46] Immenschuh S., Baumgart-Vogt E., Mueller S. (2010). Heme oxygenase-1 and iron in liver inflammation: a complex alliance. *Current Drug Targets*.

[47] Alvarez J. A., Zughaier S. M., Law J., Hao L., Wasse H., Ziegler T. R., Tangpricha V. (2013). Effects of high-dose cholecalciferol on serum markers of inflammation and immunity in patients with early chronic kidney disease. *European Journal of Clinical Nutrition*.

[48] Grossmann R. E., Zughaier S. M., Liu S., Lyles R. H., Tangpricha V. (2012). Impact of vitamin D supplementation on markers of inflammation in adults with cystic fibrosis hospitalized for a pulmonary exacerbation. *European Journal of Clinical Nutrition*.

[49] Alvarez J. A., Law J., Coakley K. E. (2012). High-dose cholecalciferol reduces parathyroid hormone in patients with early chronic kidney disease: a pilot, randomized, double-blind, placebo-controlled trial. *The American Journal of Clinical Nutrition*.

[50] Zughaier S. M., Alvarez J. A., Sloan J. H., Konrad R. J., Tangpricha V. (2014). The role of vitamin D in regulating the iron-hepcidin-ferroportin axis in monocytes. *Journal of Clinical and Translational Endocrinology*.

[51] Riek A. E., Oh J., Bernal-Mizrachi C. (2013). 1,25(OH)_2_ vitamin D suppresses macrophage migration and reverses atherogenic cholesterol metabolism in type 2 diabetic patients. *The Journal of Steroid Biochemistry and Molecular Biology*.

[52] Riek A. E., Oh J., Sprague J. E., Timpson A., De Las Fuentes L., Bernal-Mizrachi L., Schechtman K. B., Bernal-Mizrachi C. (2012). Vitamin D suppression of endoplasmic reticulum stress promotes an antiatherogenic monocyte/macrophage phenotype in type 2 diabetic patients. *Journal of Biological Chemistry*.

[53] Leon C. G., Tory R., Jia J., Sivak O., Wasan K. M. (2008). Discovery and development of toll-like receptor 4 (TLR4) antagonists: a new paradigm for treating sepsis and other diseases. *Pharmaceutical Research*.

[54] El-Achkar T. M., Hosein M., Dagher P. C. (2008). Pathways of renal injury in systemic gram-negative sepsis. *European Journal of Clinical Investigation*.

[55] Deegan S., Saveljeva S., Gorman A. M., Samali A. (2013). Stress-induced self-cannibalism: on the regulation of autophagy by endoplasmic reticulum stress. *Cellular and Molecular Life Sciences*.

[56] Cairo G., Recalcati S., Mantovani A., Locati M. (2011). Iron trafficking and metabolism in macrophages: contribution to the polarized phenotype. *Trends in Immunology*.

[57] Corna G., Campana L., Pignatti E. (2010). Polarization dictates iron handling by inflammatory and alternatively activated macrophages. *Haematologica*.

[58] Sindrilaru A., Peters T., Wieschalka S., Baican C., Baican A., Peter H., Hainzl A., Schatz S., Qi Y., Schlecht A., Weiss J. M., Wlaschek M., Sunderkötter C., Scharffetter-Kochanek K. (2011). An unrestrained proinflammatory M1 macrophage population induced by iron impairs wound healing in humans and mice. *The Journal of Clinical Investigation*.

[59] Peyssonnaux C., Zinkernagel A. S., Datta V., Lauth X., Johnson R. S., Nizet V. (2006). TLR4-dependent hepcidin expression by myeloid cells in response to bacterial pathogens. *Blood*.

[60] Abergel R. J., Clifton M. C., Pizarro J. C. (2008). The siderocalin/enterobactin interaction: a link between mammalian immunity and bacterial iron transport. *Journal of the American Chemical Society*.

[61] Bahmani P., Halabian R., Rouhbakhsh M., Roushandeh A. M., Masroori N., Ebrahimi M., Samadikuchaksaraei A., Shokrgozar M. A., Roudkenar M. H. (2010). Neutrophil gelatinase-associated lipocalin induces the expression of heme oxygenase-1 and superoxide dismutase 1, 2. *Cell Stress and Chaperones*.

[62] Yndestad A., Landrø L., Ueland T. (2009). Increased systemic and myocardial expression of neutrophil gelatinase-associated lipocalin in clinical and experimental heart failure. *European Heart Journal*.

[63] Zhang H., Xu L., Xiao D., Xie J., Zeng H., Wang Z., Zhang X., Niu Y., Shen Z., Shen J., Wu X., Li E. (2007). Upregulation of neutrophil gelatinase-associated lipocalin in oesophageal squamous cell carcinoma: significant correlation with cell differentiation and tumour invasion. *Journal of Clinical Pathology*.

[64] Zughaier S. M., Tangpricha V., Leong T., Stecenko A. A., McCarty N. A. (2013). Peripheral monocytes derived from patients with cystic fibrosis and healthy donors secrete ngal in response to Pseudomonas aeruginosa infection. *Journal of Investigative Medicine*.

[65] Warszawska J. M., Gawish R., Sharif O., Sigel S., Doninger B., Lakovits K., Mesteri I., Nairz M., Boon L., Spiel A., Fuhrmann V., Strobl B., Müller M., Schenk P., Weiss G., Knapp S. (2013). Lipocalin 2 deactivates macrophages and worsens pneumococcal pneumonia outcomes. *Journal of Clinical Investigation*.

[66] Kawasaki N., Asada R., Saito A., Kanemoto S., Imaizumi K. (2012). Obesity-induced endoplasmic reticulum stress causes chronic inflammation in adipose tissue. *Scientific Reports*.

[67] Gotoh T., Endo M., Oike Y. (2011). Endoplasmic reticulum stress-related inflammation and cardiovascular diseases. *International Journal of Inflammation*.

[68] Zhang H., Nakajima S., Kato H. (2013). Selective, potent blockade of the IRE1 and ATF6 pathways by 4-phenylbutyric acid analogues. *British Journal of Pharmacology*.

[69] Wang F.-D., Zhou D.-B., Li S.-L., Wang X., Zhang J.-P., Duan M.-H., Shen T., Wu Y.-J. (2011). Effect of recombinant human erythropoietin on hepcidin mRNA expression in patients with multiple myeloma. *Zhongguo Shi Yan Xue Ye Xue Za Zhi*.

[70] Pecoits-Filho R., Bárány P., Lindholm B., Heimbürger O., Stenvinkel P. (2002). Interleukin-6 is an independent predictor of mortality in patients starting dialysis treatment. *Nephrology Dialysis Transplantation*.

[71] McDonald B., Moyo S., Gabaitiri L., Gaseitsiwe S., Bussmann H., Koethe J. R., Musonda R., Makhema J., Novitsky V., Marlink R. G., William Wester C., Essex M. (2013). Persistently elevated serum interleukin-6 predicts mortality among adults receiving combination antiretroviral therapy in Botswana: results from a clinical trial. *AIDS Research and Human Retroviruses*.

[72] Andrié R. P., Becher U. M., Frommold R., Tiyerili V., Schrickel J. W., Nickenig G., Schwab J. O. (2012). Interleukin-6 is the strongest predictor of 30-day mortality in patients with cardiogenic shock due to myocardial infarction. *Critical Care*.

[73] Erlandson K. M., Allshouse A. A., Jankowski C. M. (2013). Association of functional impairment with inflammation and immune activation in HIV type 1-infected adults receiving effective antiretroviral therapy. *The Journal of Infectious Diseases*.

[74] Zhang J., Wu Y., Zhang Y., Leroith D., Bernlohr D. A., Chen X. (2008). The role of lipocalin 2 in the regulation of inflammation in adipocytes and macrophages. *Molecular Endocrinology*.

[75] Dixon B. M., Barker T., McKinnon T., Cuomo J., Frei B., Borregaard N., Gombart A. F. (2012). Positive correlation between circulating cathelicidin antimicrobial peptide (hCAP18/LL-37) and 25-hydroxyvitamin D levels in healthy adults. *BMC Research Notes*.

[76] Wiwanitkit V. (2006). Quantum chemical analysis of the deferiprone-iron binding reaction. *International Journal of Nanomedicine*.

[77] Nurchi V. M., Crisponi G., Pivetta T., Donatoni M., Remelli M. (2008). Potentiometric, spectrophotometric and calorimetric study on iron(III) and copper(II) complexes with 1,2-dimethyl-3-hydroxy-4-pyridinone. *Journal of Inorganic Biochemistry*.

[78] Kontoghiorghes G. J., Spyrou A., Kolnagou A. (2010). Iron chelation therapy in hereditary hemochromatosis and thalassemia intermedia: regulatory and non regulatory mechanisms of increased iron absorption. *Hemoglobin*.

[79] Fischbach M. A., Lin H., Liu D. R., Walsh C. T. (2006). How pathogenic bacteria evade mammalian sabotage in the battle for iron. *Nature Chemical Biology*.

[80] Chang H. T., Olson L. K., Schwartz K. A. (2013). Ketolytic and glycolytic enzymatic expression profiles in malignant gliomas: implication for ketogenic diet therapy. *Nutrition and Metabolism*.

