# Supplementary material for: Inflammation and ER Stress Downregulate BDH2 Expression and Dysregulate Intracellular Iron in Macrophages
Source: J Immunol Res. 2014 Dec 1;2014:140728. doi: 10.1155/2014/140728 (PMC4267003; doi:10.1155/2014/140728)
Supplement: Supplementary file 1 — Supplementary Figure 1: Cellular viability and iron retention in tunicamycin treated macrophages. A: Cellular viability of THP-1 cells treated with tunicamycin doses prior to LPS exposure was assessed using the MTT assay (MTT OD-A591). B: THP-1 cells (1x10^6^ cell/ml) were treated with tunicamycin doses or DMSO overnight prior to LPS exposure (40 ng/ml) for 6 hr and iron retention in macrophages was determined using calcein-AM. DMSO treated cells were incubated simultaneously and used as controls. Calcein-AM fluorescence is quenched upon binding iron and is therefore inversely correlated with intracellular iron accumulation. AU: arbitrary units. Error bars represent the SD from the mean of triplicate readings and data are representative of two independent experiments. [file 140728.f1.pdf]

Inflammation and ER stress downregulate BDH2 expression and dysregulate intracellular iron in macrophages

Susu M. Zughaier*[1†], Brandon B. Stauffer[2], Nael A. McCarty[2]

Supplementary Figures:

Supplementary Figure 1: Cellular viability and iron retention in tunicamycin treated macrophages. A: Cellular viability of THP-1 cells treated with tunicamycin doses prior to LPS exposure was assessed using the MTT assay (MTT OD-A591). **B**: THP-1 cells ($1x10^6$ cell/ml) were treated with tunicamycin doses or DMSO overnight prior to LPS exposure (40 ng/ml) for 6 hr and iron retention in macrophages was determined using calcein-AM. DMSO treated cells were incubated simultaneously and used as controls. Calcein-AM fluorescence is quenched upon binding iron and is therefore inversely correlated with intracellular iron accumulation. AU: arbitrary units. Error bars represent the SD from the mean of triplicate readings and data are representative of two independent experiments.

A

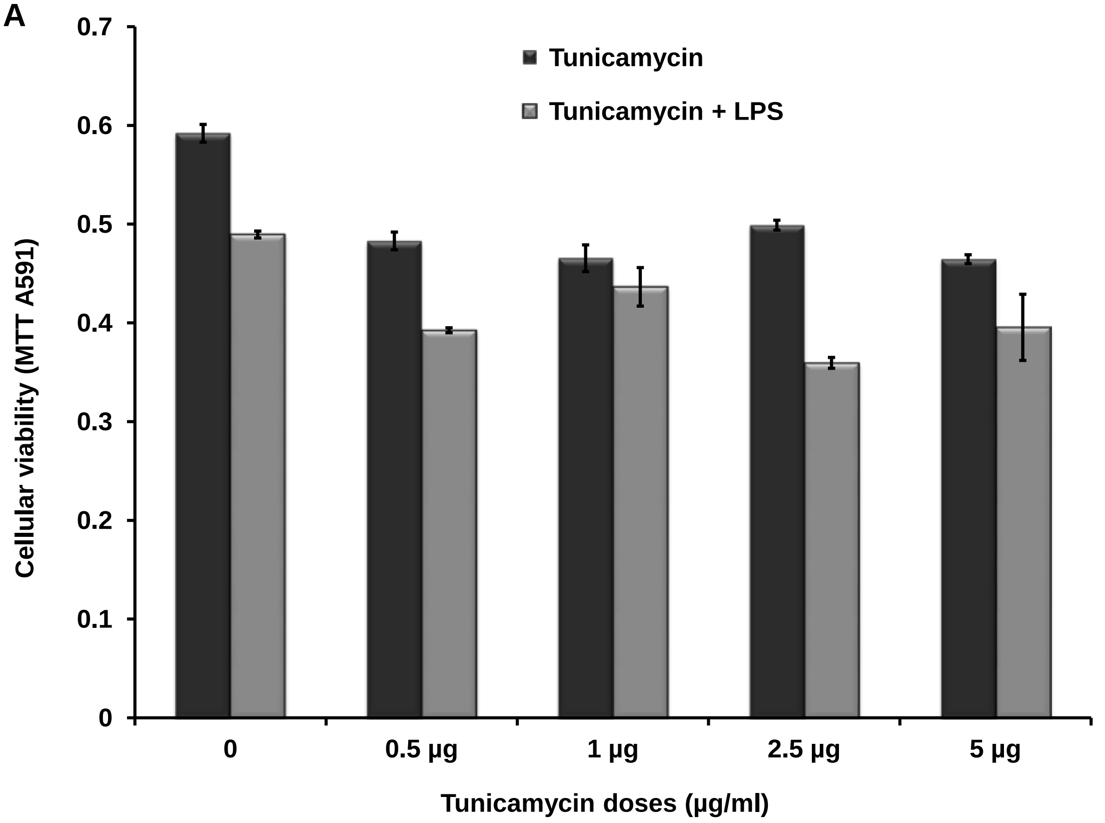

B

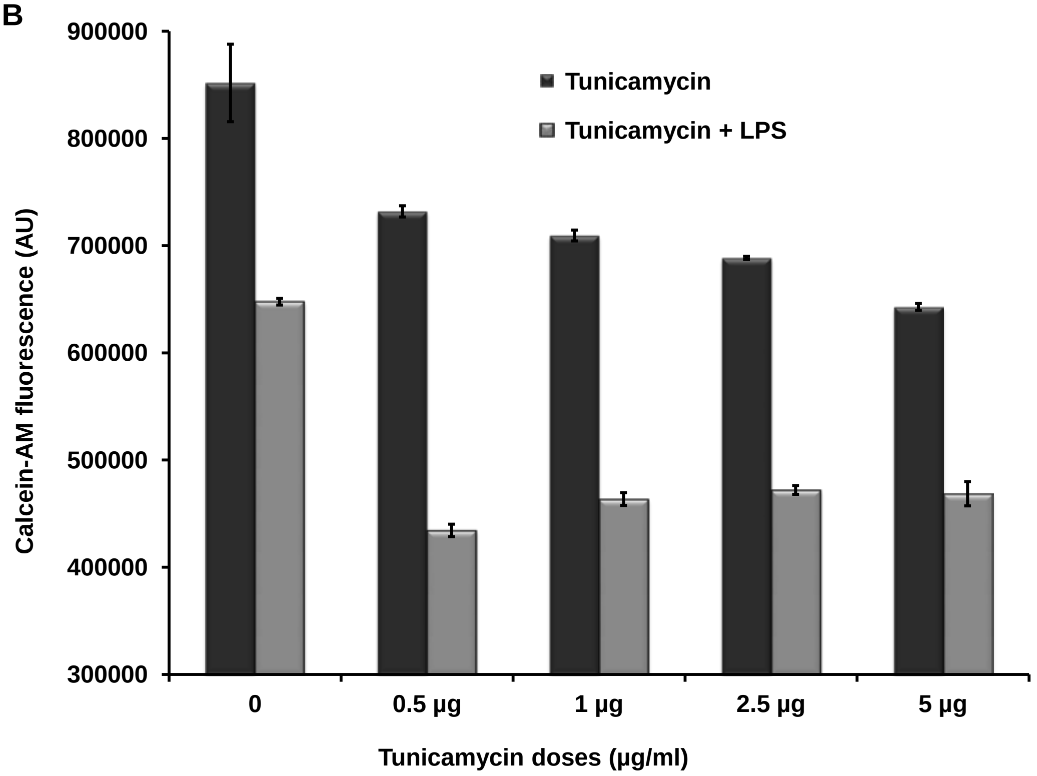
900000
800000
700000
600000
500000
400000
300000
Calcein-AM fluorescence (AU)
Tunicamycin
Tunicamycin + LPS
0
0.5 µg
1 µg
2.5 µg
5 µg
Tunicamycin doses (µg/ml)
